# Palliative care in hematologic malignancies: a bibliometric review and visualization analysis from 2000 to 2025

**DOI:** 10.3389/fonc.2026.1939505

**Published:** 2026-10-16

**Authors:** Xiaofeng Zeng, Jiatong Yao, Li Mo, Xiaoli Huang

**Affiliations:** 1 Center of Gerontology and Geriatrics, National Clinical Research Center for Geriatrics, West China Hospital, Sichuan University, Chengdu, China; 2 Health Management Center of West China Hospital, Sichuan University, Chengdu, China; 3 Department of Gastroenterology and Hepatology, West China Hospital, Sichuan University, Chengdu, China

**Keywords:** bibliometric analysis, health care access, hematologic malignancies, hematopoietic stem-cell transplantation, hospice care, palliative care, quality of life

## Abstract

**Introduction:**

Patients with hematologic malignancies experience a substantial symptom burden and uncertain disease trajectories, yet palliative care is often integrated later than in solid-tumor oncology. This study characterized publication patterns, collaboration networks, research topics, and citation structures in this literature from 2000 to 2025.

**Methods:**

The Web of Science Core Collection and Scopus were searched on June 10, 2026, for English-language articles and reviews published during 2000-2025. After database integration, deduplication, screening, and full-text treatment-content verification, 307 publications were included. VOSviewer and the bibliometrix package in R were used to summarize annual output, leading contributors, keyword co-occurrence, trend topics, thematic communities, keyword bursts, local citations, and reference co-citation. A structured narrative synthesis contextualized highly cited and recent publications.

**Results:**

The corpus comprised 258 articles and 49 reviews published between 2001 and 2025. Annual output increased at a descriptive rate of 17.6% and was higher after 2017, reaching 49 publications in 2025. The United States contributed 146 publications, while international co-authorship accounted for 14.98% of the corpus. Duke University and Harvard University were the leading institutional groups, Thomas W. LeBlanc was the most productive author, and the Journal of Palliative Medicine published the most articles. Seven palliative, supportive care, or hospice journals accounted for 38.8% of publications, whereas three leading hematology journals contributed 19 articles. Keyword co-occurrence, median publication years, thematic communities, and burst analyses indicated that recent publications more frequently addressed early palliative care, integration, communication, quality of life, health care access, equity and disparities, and care alongside hematopoietic stem-cell transplantation. The substantive literature described late referral, intensive care near death, transfusion access, prognostic uncertainty, caregiver needs, and limited primary palliative care capacity.

**Conclusions:**

This literature has expanded and increasingly examines concurrent palliative care during intensive hematologic treatment. Research remains concentrated in a limited number of countries, institutions, author groups, and palliative care journals. Further empirical studies could examine underrepresented settings and topics, including community, rural, and international care, caregiver support, primary palliative care, equity, and palliative transfusion. These bibliometric findings describe the structure and topics of the literature; they do not address clinical effectiveness or changes in practice.

## 1 Introduction

Hematologic malignancies are frequently characterized by relapsing, rapidly evolving, and unpredictable disease trajectories. Repeated intensive therapy, cytopenia-related complications, and infections can impose substantial physical and psychological symptom burden (1, 2). Since hematologic malignancies may remain treatment-sensitive even at advanced stages, potentially curative or life-prolonging therapy may remain appropriate late in the disease course, making the transition to comfort-focused care less clear (1). Therefore, palliative care holds a distinctive role in hematology. It relieves symptoms, improves quality of life, supports patients and their families, and facilitates decision-making that aligns with individual goals (3). A randomized trial in hematopoietic cell transplantation demonstrated that inpatient palliative care attenuated early deterioration in quality of life and limited the worsening of psychological symptoms (4). This evidence is reflected in the 2024 American Society of Clinical Oncology (ASCO) guideline, which recommends early, concurrent specialist palliative care for patients with advanced cancer, including those with hematologic malignancies (5). Guidance from the National Comprehensive Cancer Network and educational literature from the American Society of Hematology similarly support needs-based, disease-adapted integration (6, 7). Despite this rationale, palliative care integration remains less routine and often occurs later in hematologic than in solid-tumor oncology (8, 9).

Integrating palliative care into hematologic oncology remains challenging because clinician-related, disease- and treatment-related, and health-system barriers often intersect. Clinicians must balance uncertain prognoses and the potential benefits of further disease-directed therapy against timely discussions of symptom relief and end-of-life planning (9, 10). Some clinicians also associate palliative care with terminal care or worry that referral may diminish hope or disrupt long-standing therapeutic relationships (9, 11). Rapid, unpredictable deterioration and continued dependence on transfusions, antimicrobial therapy, or other life-sustaining measures further complicate referral and hospice enrollment (12, 13). Restrictive eligibility criteria, limited access, and uneven service availability may therefore force patients to choose between ongoing treatment and hospice, particularly in rural or resource-limited settings (14, 15). These constraints also highlight the need to strengthen primary palliative care capacity within hematology while maintaining access to specialist services (11, 16). Although studies in transplantation and other hematologic settings have demonstrated benefits from specialist palliative care, referral often remains late (4, 16). Thus, the field is clinically important but remains heterogeneous in its concepts, delivery models, and access pathways.

Conventional reviews are valuable for evaluating specific interventions and outcomes, but they offer limited insight into the broader development and collaborative structure of a research field. Bibliometric analysis and visual knowledge mapping can complement these approaches by revealing publication trends, influential contributors, collaboration patterns, knowledge structures, and emerging research themes (17). Accordingly, this study mapped research on palliative care in hematologic malignancies published between 2000 and 2025. We examined publication trends, identified leading countries, institutions, journals, and authors, and assessed collaboration patterns together with thematic evolution. By clarifying the development of this field and its research priorities, this analysis may inform future studies and support the integration of palliative care into hematology practice.

## 2 Materials and methods

This study adhered to the BIBLIO checklist, the preliminary guideline for reporting bibliometric reviews of the biomedical literature (18) (**Supplementary Table S1**). The methodological framework and reporting of this bibliometric analysis were refined according to the VALOR (Evaluating Bibliometrics Reviews) practical guide (19).

### 2.1 Data sources and search strategy

Bibliographic records were retrieved from the Web of Science Core Collection (WoSCC) and Scopus. The search was run on June 10, 2026, and covered publications dated from January 1, 2000, to December 31, 2025. Only English-language articles and reviews were retained. The search strategy combined hematologic malignancy and palliative care terms, adapted from previous systematic reviews (20, 21). Broad truncation of palliative* can retrieve records in which “palliative” refers only to non-curative chemotherapy, radiotherapy, cytoreduction, or another cancer-directed modality. A prespecified treatment-content eligibility screen was therefore applied after the general eligibility screen (Section 2.3). “Supportive care” was not used as a standalone search term, because it encompasses a broader literature on treatment toxicity, infection prophylaxis, transfusion support, rehabilitation, and survivorship that extends beyond the intended palliative care focus.

For WoSCC, the search strategy was formulated as follows: TS=(“Hematologic* Neoplasm*” OR “Haematologic* Neoplasm*” OR “hematolog* malignan*” OR “haematolog* malignan*” OR “myeloid neoplas*” OR “lymphoid neoplas*” OR leukemia* OR leukaemia* OR lymphoma* OR hodgkin* OR non-hodgkin* OR “multiple myeloma*” OR “plasma cell neoplas*” OR “plasma cell dyscrasia*” OR myelodysplas* OR myeloproliferative OR “myeloid* sarcoma*”) AND TS=(“palliative care” OR “palliative medicine” OR “palliative nursing” OR “palliative therapy” OR palliation OR palliative* OR hospice* OR “end of life*” OR “end-of-life” OR “terminal care” OR “terminally ill*” OR “advance* care”).

For Scopus, the search strategy was formulated as follows: TITLE-ABS-KEY(“Hematologic* Neoplasm*” OR “Haematologic* Neoplasm*” OR “hematolog* malignan*” OR “haematolog* malignan*” OR “myeloid neoplas*” OR “lymphoid neoplas*” OR leukemia* OR leukaemia* OR lymphoma* OR hodgkin* OR non-hodgkin* OR “multiple myeloma*” OR “plasma cell neoplas*” OR “plasma cell dyscrasia*” OR myelodysplas* OR myeloproliferative OR “myeloid* sarcoma*”) AND TITLE-ABS-KEY(“palliative care” OR “palliative medicine” OR “palliative nursing” OR “palliative therapy” OR palliation OR palliative* OR hospice* OR “end of life*” OR “end-of-life” OR “terminal care” OR “terminally ill*” OR “advance* care”).

### 2.2 Eligibility criteria

Records were eligible if they (1) addressed palliative care, hospice care, end-of-life care, terminal care, advance care planning, or related supportive care issues in hematologic malignancies; (2) were classified as an article or review; (3) were published in English; and (4) were published between 2000 and 2025. Records were excluded if they were unrelated to palliative care in hematologic malignancies, were case reports or study protocols, focused on children or adolescents, primarily concerned solid tumors such as lung or breast cancer, or involved animal studies.

### 2.3 Data integration, deduplication, and screening

Raw WoSCC and Scopus records were integrated into a harmonized data structure using Python (version 3.10.4) and R (version 4.4.2). Core bibliographic fields were standardized across database-specific formats. Author, affiliation, keyword, cited-reference, and citation data were then consolidated before deduplication and bibliometric analysis.

After field harmonization, records were deduplicated computationally. Duplicate records were identified using DOI when available. When DOI information was missing or inconsistent, duplicates were identified by matching normalized titles, first authors, and publication years. Potential duplicates were verified manually before removal.

Deduplicated records were screened independently by two reviewers (XFZ and JTY) using titles and abstracts. Disagreements were resolved by discussion, with unresolved cases adjudicated by a senior reviewer (XLH). Records retained after this general screen underwent full-text verification incorporating the prespecified treatment-content screen. Occurrences of “palliative” or “palliation” were evaluated against treatment-modality and treatment-intent terms. A record was excluded only when the palliative term referred solely to non-curative antineoplastic treatment and the record contained no substantive content on palliative, hospice, end-of-life, symptom or supportive care; communication or decision support; or caregiver support.

### 2.4 Bibliometric and visualization analysis

Bibliometric analyses were performed with VOSviewer (version 1.6.20) (22) and the bibliometrix package in R (version 4.4.2) (23). Bibliometrix summarized annual publication output and leading countries, institutional groups, authors, and source titles. Publication counts by country, institution, and author were based on whole counting: a publication involving several entities was counted once for each entity, so entity counts exceed 307 in total. VOSviewer maps displayed the 10 most productive countries and 16 authors with at least six publications. Node size represented publication output, link thickness indicated collaboration strength, and color denoted cluster membership.

Author and indexed keywords were harmonized for synonyms, spelling variants, and singular/plural forms; non-specific terms were removed using a stop-word list. The structural and overlay maps retained keywords present in at least 10 documents. Node size indicated document-level occurrence, link thickness indicated co-occurrence strength, cluster color denoted community membership, and overlay color denoted the average publication year.

The trend topics analysis considered harmonized concepts present in at least two documents. For each concept, the document-level median publication year and interquartile range (IQR) were plotted, with point size indicating document frequency. Thematic evolution was evaluated across four prespecified slices (2000-2013, 2014-2017, 2018-2021, and 2022-2025), chosen to accommodate publication growth while retaining four-year resolution in recent years. Within each slice, binary document-level co-occurrence networks were constructed from up to 60 terms occurring in at least two documents, Louvain communities were identified, and adjacent-period communities were linked when they shared terms. Keyword bursts were detected for terms occurring in at least three documents. A Kleinberg-style finite-state automaton with data-determined burst levels (s = 2, γ = 0.3) (24) was applied to yearly counts normalized by the annual number of documents. Burst strength was the accumulated cost advantage of the elevated state. Bursts ending in 2021 or later were retained, and the 30 strongest are reported.

Local citations were defined as citations received from other records within the final corpus. Reference co-citation analysis characterized the field’s intellectual structure. The network was constructed from the 40 most frequently cited references, retaining co-citation links observed at least twice; after removal of isolated nodes, 39 cited-reference nodes remained. Communities were identified with the Louvain algorithm. PageRank (damping factor 0.85), betweenness centrality, and closeness centrality were computed with igraph routines on the undirected weighted network.

### 2.5 Narrative synthesis of substantive literature

Bibliometric indicators describe patterns of terminology and association; they do not capture clinical content. We therefore complemented the quantitative mapping with a structured narrative synthesis of substantive publications, following established guidance on narrative synthesis in systematic reviews (25). Highly locally cited and recent publications corresponding to the burst terms and late-emerging themes identified in the keyword, trend topic, and thematic evolution analyses were read in full. Two reviewers (XFZ and JTY) independently read each publication and extracted the study design, population, setting, and principal findings into a structured template. Disagreements were resolved by discussion, with unresolved cases adjudicated by a senior reviewer (XLH). Extracted findings were grouped into thematic strands and compared with the corresponding bibliometric signals.

## 3 Results

### 3.1 Search results, general characteristics, and annual publication trends

The searches identified 9,072 records (WoSCC, 2,349; Scopus, 6,723). Removal of 1,848 duplicates left 7,224 records for title/abstract screening, of which 6,863 were excluded. The treatment-content screen excluded a further 54 records (37 local symptom-directed treatment records, 16 non-curative systemic-treatment records, and one palliative-surgery record), yielding 307 included publications (258 articles and 49 reviews; **Figure 1**). The publications spanned 2001 to 2025 and appeared in 108 harmonized source titles; the corpus contained 11,798 cited references and 1,615 authors. The mean number of co-authors per document was 6.85, and international co-authorship accounted for 14.98% (**Supplementary Table S2**). Annual publication output increased over the observation period, with a descriptive annual growth rate of 17.6% (**Figure 2**; **Supplementary Table S2**). Annual output remained at five or fewer publications through 2013, first reached 10 in 2014, rose to 19 in 2017, and increased to 35 in 2024 and 49 in 2025.

**Figure 1:**
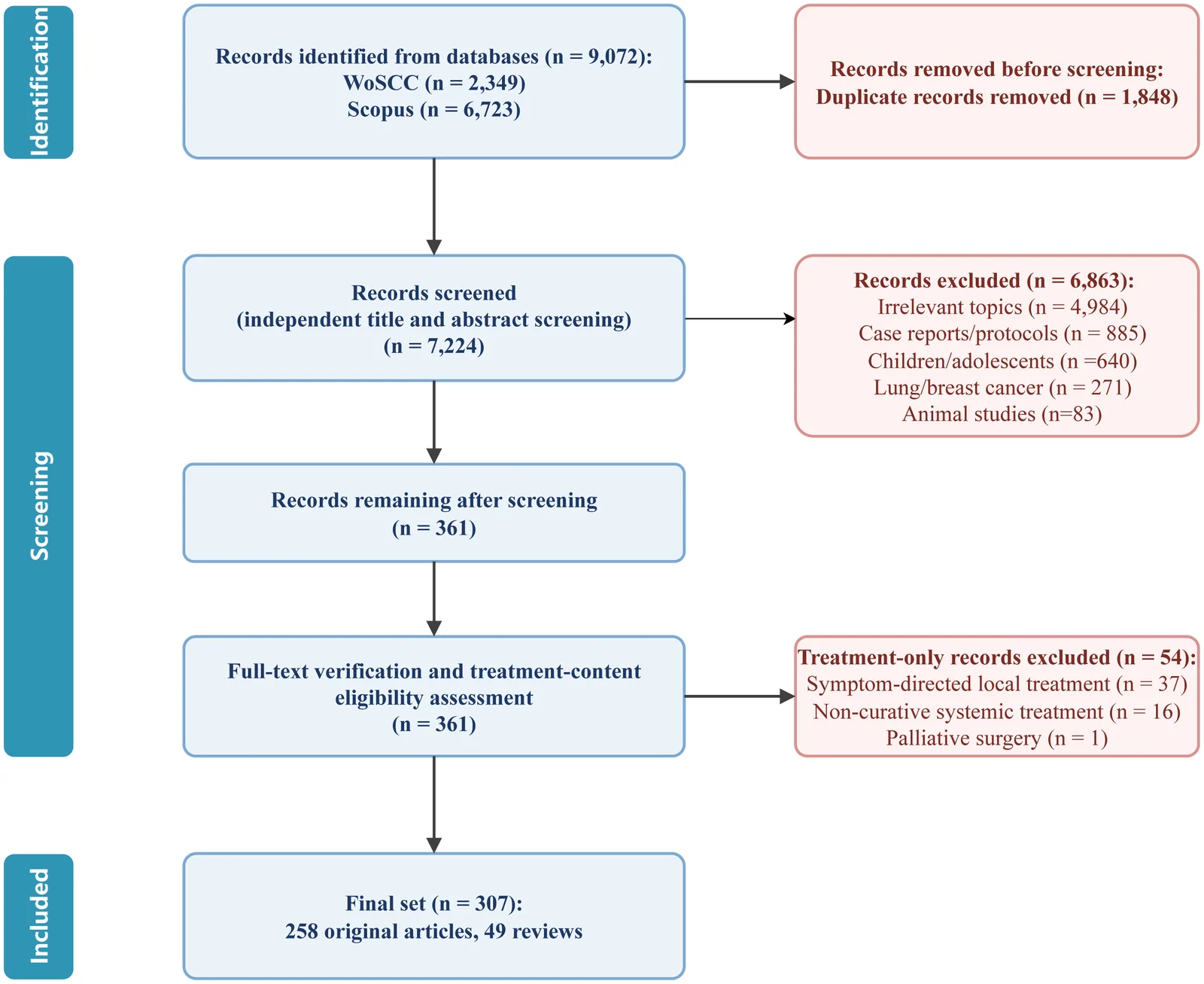
Bibliometric record selection flow diagram. Records on palliative care in hematologic malignancies were identified from the Web of Science Core Collection and Scopus. The final dataset retained for bibliometric analysis included 307 records, comprising 258 articles and 49 reviews.

**Figure 2:**
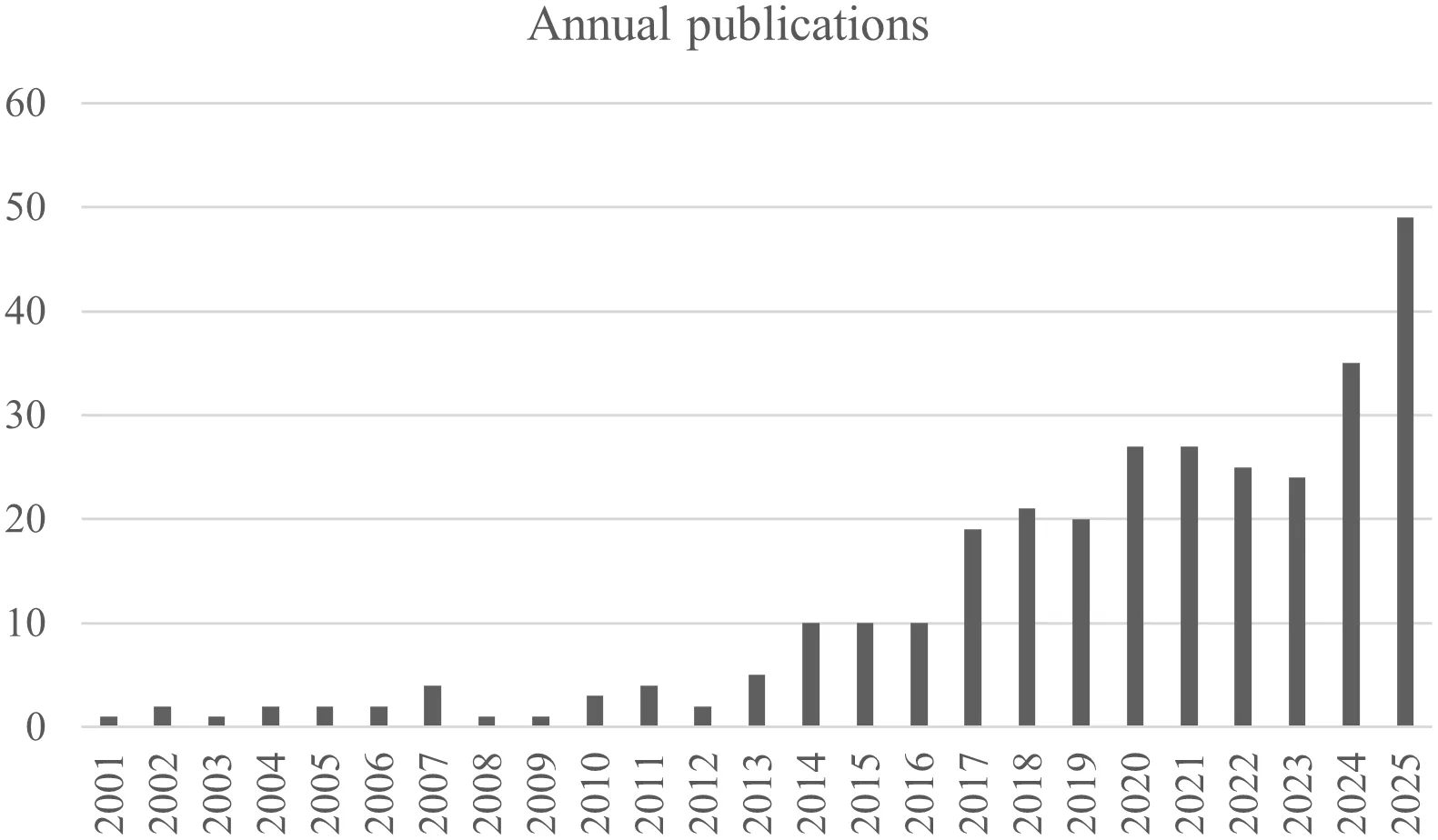
Annual publication trends of research on palliative care in hematologic malignancies from 2000 to 2025.

### 3.2 Productive countries, institutions, authors, and journals

The United States was the leading contributor (146 publications; 4,729 citations), followed by the United Kingdom (28 publications; 1,208 citations) and Canada (21 publications) (**Supplementary Table S3**). In the country collaboration network, the United States was the largest and most connected node; strong collaborative links were also evident for Canada, Italy, and the United Kingdom, whereas China showed comparatively few links, and Japan showed no visible link at the applied threshold (**Figure 3**).

**Figure 3:**
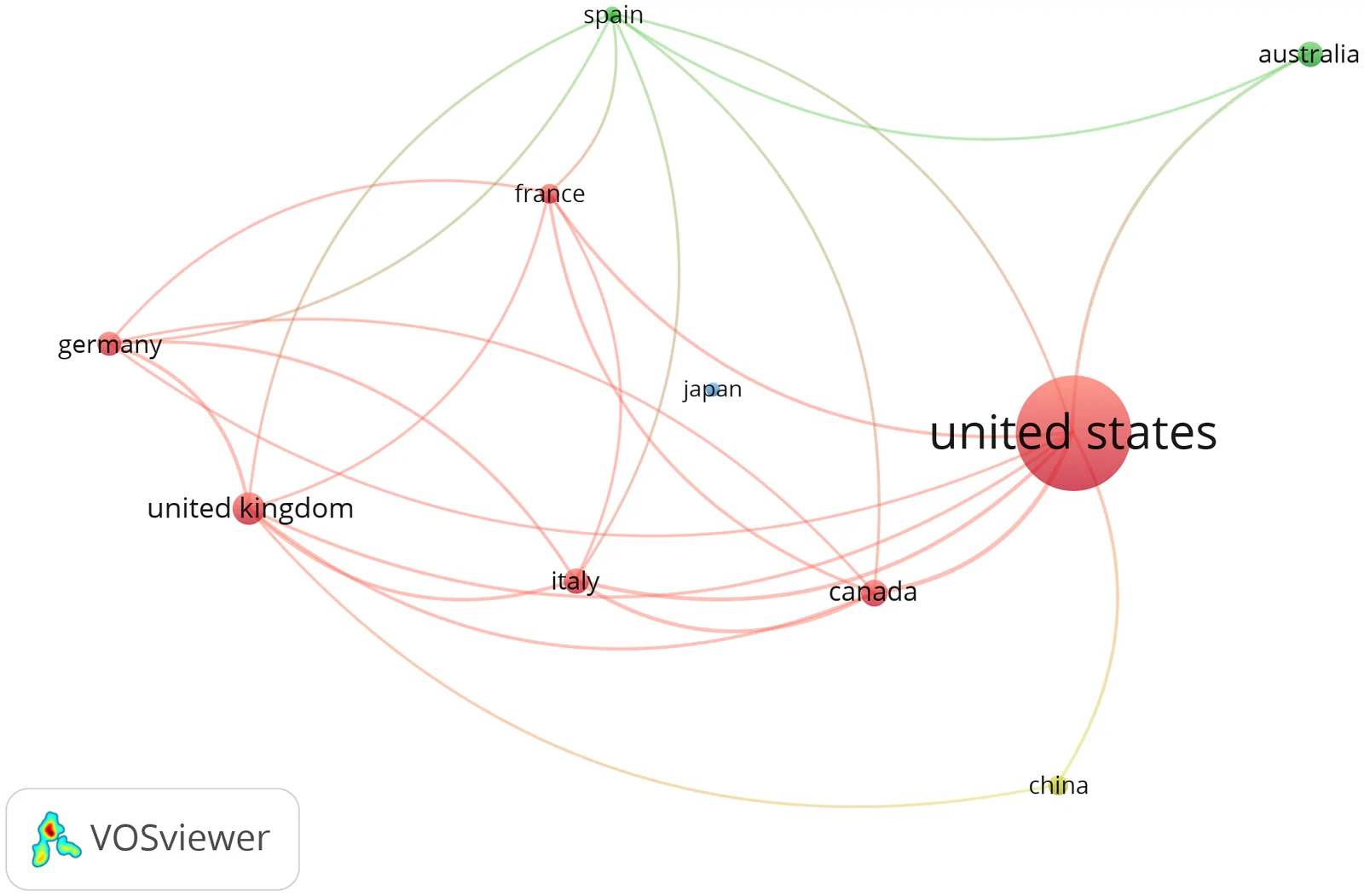
Country collaboration network. The network was mapped using VOSviewer. Nodes represent individual countries, with node size proportional to the total publication output. The thickness of the connecting links indicates the strength of collaboration between countries, and colors differentiate collaborative clusters. The network highlights the United States as the largest and most central node.

The Duke University group ranked first by output (42 publications; 2,206 citations), followed by the Harvard University group (38 publications; 2,063 citations) and the University of Toronto group (11 publications) (**Table 1**). These labels follow the predefined institutional aggregation used in the analysis: the Duke group includes Duke University and Duke Cancer Institute, and the Harvard group includes Harvard University, Harvard Medical School, Massachusetts General Hospital, Dana-Farber Cancer Institute, and Brigham and Women’s Hospital. Group totals should therefore not be interpreted as like-for-like comparisons of single-university output.

**Table 1:** Leading organizations contributing to palliative care research in hematologic malignancies.

Organization	Publications	Citations	Citations/publications	Country	Productive authors
Duke University group	42	2,206	52.52	United States	Thomas W. LeBlanc; Jason A. Webb; Neha Kayastha
Harvard University group	38	2,063	54.29	United States	Oreofe O. Odejide; Areej El-Jawahri; Gregory A. Abel
University of Toronto group	11	192	17.45	Canada	Camilla Zimmermann; Gary Rodin
University of Washington group	9	769	85.44	United States	Anthony L. Back; Stephanie J. Lee
University of York	9	606	67.33	United Kingdom	Debra A. Howell; Eve Roman; Alexandra G. Smith
The University of Texas MD Anderson Cancer Center	7	447	63.86	United States	Eduardo Bruera; David Hui
Memorial Sloan Kettering Cancer Center	7	34	4.86	United States	Judith E. Nelson; Miguel-Angel Perales; Anjali V. Desai
Castle Hill Hospital	6	452	75.33	United Kingdom	Russell D. Patmore
University of Modena and Reggio Emilia	6	94	15.67	Italy	Leonardo Potenza; Mario Luppi; Eleonora Borelli
Oregon Health & Science University	6	51	8.50	United States	Jason A. Webb

Duke University group: Duke University; Duke Cancer Institute. Harvard University group: Harvard University; Harvard Medical School; Massachusetts General Hospital (MGH); Dana-Farber Cancer Institute; Brigham and Women's Hospital. University of Toronto group: University of Toronto; University Health Network. University of Washington group: University of Washington; Fred Hutchinson Cancer Research Center.

Thomas W. LeBlanc was the most productive author (35 publications; 1,789 citations), followed by Areej El-Jawahri (15 publications; 1,174 citations), and Eduardo Bruera (12 publications; 641 citations) (**Table 2**). Among the authors listed in **Table 2**, Jennifer S. Temel had the highest citations per publication (150.17). The co-authorship map showed three connected clusters; one displayed author, Eve Roman, was isolated (**Supplementary Figure S1**). The topics described for these authors in **Table 2** included integrated palliative care during acute myeloid leukemia (AML) treatment and hematopoietic stem-cell transplantation (HSCT), symptom and psychological outcomes, communication, hospice use, and home-based care.

**Table 2:** Leading authors contributing to palliative care research in hematologic malignancies.

Author	Publications	Citations	Citations/publications	Country	Affiliations	Main research areas
Thomas W. LeBlanc	35	1,789	51.11	United States	Duke University group	Early and integrated palliative care for hematologic malignancies, AML, HSCT, symptom burden, quality of life, hospice, and end-of-life care.
Areej El-Jawahri	15	1,174	78.27	United States	Harvard University group	Integrated palliative-oncology care in AML and HSCT, inpatient palliative care interventions, psychological distress, quality of life, and caregiver outcomes.
Eduardo Bruera	12	641	53.42	United States	The University of Texas MD Anderson Cancer Center	Supportive and palliative care referral, symptom burden, end-of-life care quality, clinician attitudes, goals-of-care conversations, leukemia, and myeloma.
Oreofe O. Odejide	11	440	40.00	United States	Harvard University group	End-of-life care in blood cancers, hospice use, goals-of-care discussions, care quality, and health care utilization.
Gregory A. Abel	8	505	63.12	United States	Harvard University group	End-of-life care quality, hospice use, health services outcomes, health care utilization, and clinician communication in hematologic malignancies.
Jason A. Webb	8	257	32.12	United States	Duke University group; Oregon Health & Science University	AML-focused palliative care integration, implementation pathways, clinical guidance for palliative care clinicians, and supportive care models.
Camilla Zimmermann	8	157	19.62	Canada	University of Toronto group	Early and integrated palliative care, acute leukemia, multiple myeloma, outpatient palliative care, psychosocial intervention, quality of life, and symptom-focused care.
Stephanie J. Lee	7	715	102.14	United States	University of Washington group	HSCT, inpatient palliative care, advance care planning, quality-of-life response, psychological distress, and transplant-related supportive care.
Angel M. Cronin	6	320	53.33	United States	Harvard University group	End-of-life outcomes, hospice barriers, health services research, physician communication, prognosis discussion, and transfusion access.
Fabio Efficace	6	103	17.17	Italy	Italian Group for Adult Hematologic Diseases	Early palliative/supportive care, quality of life, patient-reported outcomes, home care, cost-effectiveness, and GIMEMA collaborative studies.
David Hui	6	419	69.83	United States	The University of Texas MD Anderson Cancer Center	Timing of palliative care referral, supportive care guidelines, symptom burden, goals-of-care programs, and clinician attitudes toward referral.
Pasquale Niscola	6	101	16.83	Italy	University of Rome Tor Vergata	Home-based supportive and palliative care, malignant hematology, and terminal care.
Leonardo Potenza	6	83	13.83	Italy	University of Modena and Reggio Emilia	Early palliative/supportive care in AML and multiple myeloma, quality of life, end-of-life aggressiveness, and GIMEMA observational studies.
Eve Roman	6	491	81.83	United Kingdom	University of York	Place of death, specialist palliative care referrals, hospice access, bereaved relatives’ perspectives, and population-based studies in hematologic malignancies.
Jennifer S. Temel	6	901	150.17	United States	Harvard University group	Integrated palliative-oncology care in AML and HSCT, psychological distress, and quality of life.
James A. Tulsky	6	318	53.00	United States	Harvard University group; Duke University group	Goals-of-care communication, prognosis discussions, hospice and transfusion access, advance care planning, and end-of-life utilization.

AML, acute myeloid leukemia; HSCT, hematopoietic stem-cell transplantation; GIMEMA, Italian Group for Adult Hematologic Diseases.

After pooling source-title variants, the 13 leading journals contributed 156 of the 307 publications (50.8%; **Table 3**). Journal of Palliative Medicine ranked first (27 publications), followed by American Journal of Hospice and Palliative Medicine (23 publications) and Supportive Care in Cancer (22 publications). Seven palliative, supportive care, or hospice journals accounted for 119 publications (38.8% of the corpus). The three hematology journals in **Table 3** contributed 19 publications, and the three oncology journals contributed 18. American Journal of Hospice and Palliative Medicine had the earliest included publication among these sources (in 2002). Cancer and Journal of Clinical Oncology had the highest citations per publication among the listed sources (102.67 and 97.17, respectively).

**Table 3:** Leading sources contributing to palliative care research in hematologic malignancies.

Source	Publications	Citations	Citations/publications	Year range in the dataset
Journal of Palliative Medicine	27	610	22.59	2008-2025
American Journal of Hospice and Palliative Medicine	23	312	13.57	2002-2025
Supportive Care in Cancer	22	276	12.55	2012-2025
Journal of Pain and Symptom Management	14	586	41.86	2011-2025
BMJ Supportive & Palliative Care	12	257	21.42	2015-2025
BMC Palliative Care	11	368	33.45	2010-2025
Palliative Medicine	10	518	51.80	2007-2023
European Journal of Haematology	7	111	15.86	2017-2025
Cancer	6	616	102.67	2014-2021
Journal of Clinical Oncology	6	583	97.17	2007-2025
Blood Advances	6	101	16.83	2019-2025
Annals of Hematology	6	99	16.50	2014-2025
Cancers	6	43	7.17	2022-2025

### 3.3 Keyword co-occurrence and thematic evolution

The co-occurrence analysis retained 45 harmonized author and indexed keywords present in at least 10 documents. “Palliative care” was the most frequent keyword (211 documents), followed by “hematologic malignancies” (181), “quality of life” (145), and “hospice care” (132) (**Table 4**). Three keyword clusters were identified (**Figure 4**). The first cluster (red) grouped disease-, treatment-, and symptom-related terms (multiple myeloma, lymphoma, chemotherapy, blood transfusion, critical care, pain, fatigue, anxiety, and depression, together with decision making, prognosis, and survival), reflecting research on disease management and symptom burden. The second cluster (green) centered on palliative care, hospice care, and end of life, together with communication, discussions, advance care planning, attitudes, needs, early palliative care, aggressiveness, and predictors, representing end-of-life care processes and clinician-patient communication. The third cluster (blue) linked quality of life, symptoms, outcomes, advanced cancer, stem-cell transplantation, supportive care, integration, consultation, and caregivers, reflecting patient-reported outcomes and the integration of palliative and supportive care.

**Table 4:** The list of high-frequency keywords (present in at least ten documents).

Keyword	Occurrences	Total link strength
palliative care	211	1202
hematologic malignancies	181	1043
quality of life	145	831
hospice care	132	820
cancer	130	762
leukemia	84	506
end of life	83	478
symptoms	69	454
multiple myeloma	49	308
stem-cell transplantation	48	330
hematology	42	259
outcomes	41	257
older adults	39	274
advanced cancer	38	243
supportive care	38	221
mortality	34	204
chemotherapy	31	212
caregivers	27	162
advance care planning	26	154
lymphoma	25	189
early palliative care	24	147
hematologic disease	24	151
pain	22	134
attitudes	20	129
predictors	20	122
cancer palliative therapy	19	143
communication	19	110
survival	19	98
consultation	18	115
aggressiveness	16	119
decision making	16	101
discussions	16	103
hospitalization	15	116
cancer care	14	88
perceptions	13	75
integration	12	90
prognosis	12	81
anxiety	11	88
critical care	11	86
depression	11	90
needs	11	70
nursing	11	76
blood transfusion	10	69
fatigue	10	76
psychology	10	80

**Figure 4:**
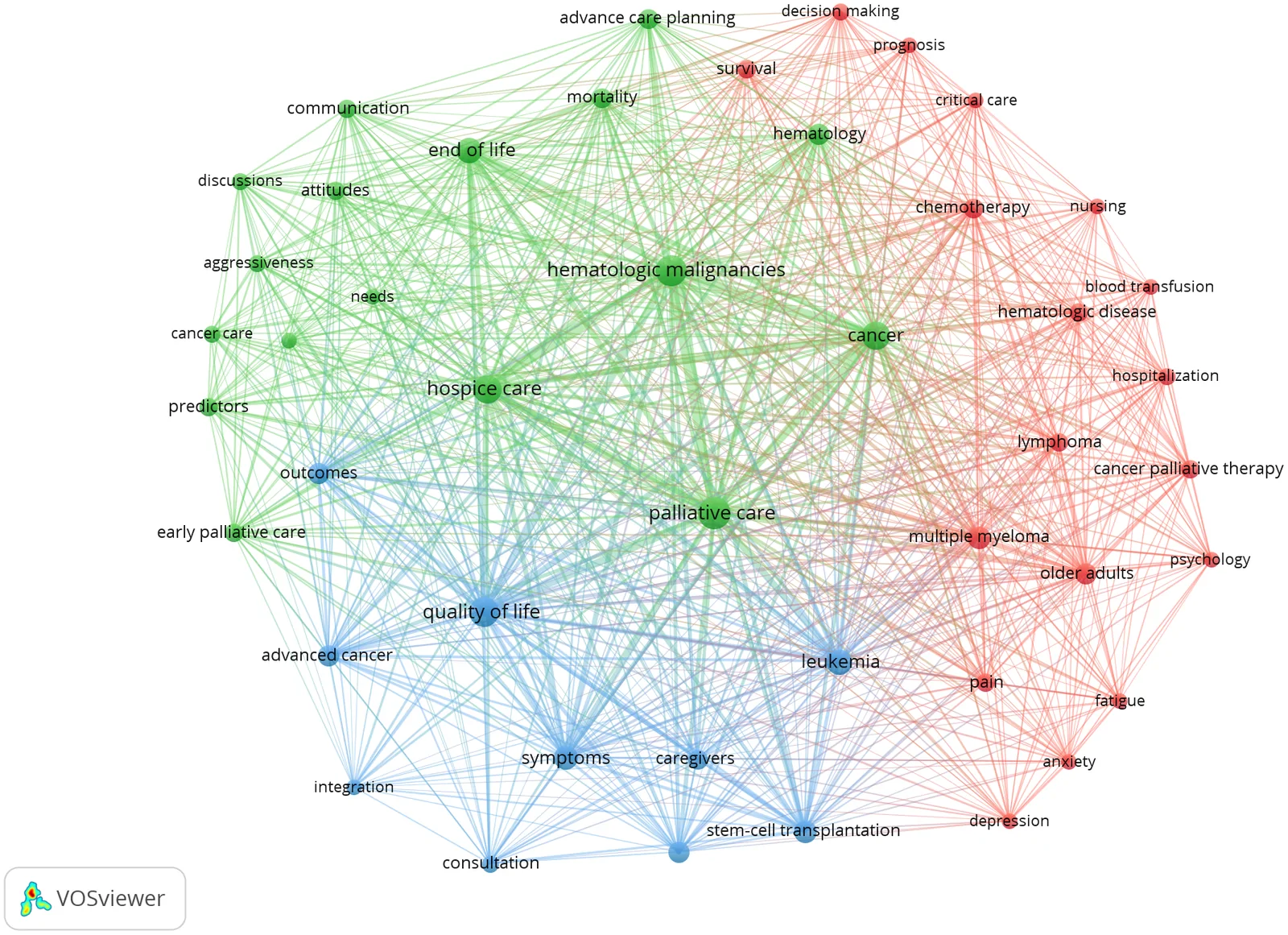
Keyword co-occurrence network. The network displays author and indexed keywords present in at least 10 documents. Node size is proportional to the occurrence frequency, line thickness indicates co-occurrence strength, and node colors denote distinct thematic clusters.

In the overlay visualization (**Figure 5**), disease-, treatment-, and prognosis-related terms appeared earliest; communication, advance care planning, quality of life, and symptoms appeared later; and early palliative care, integration, discussions, needs, and perceptions appeared most recently.

**Figure 5:**
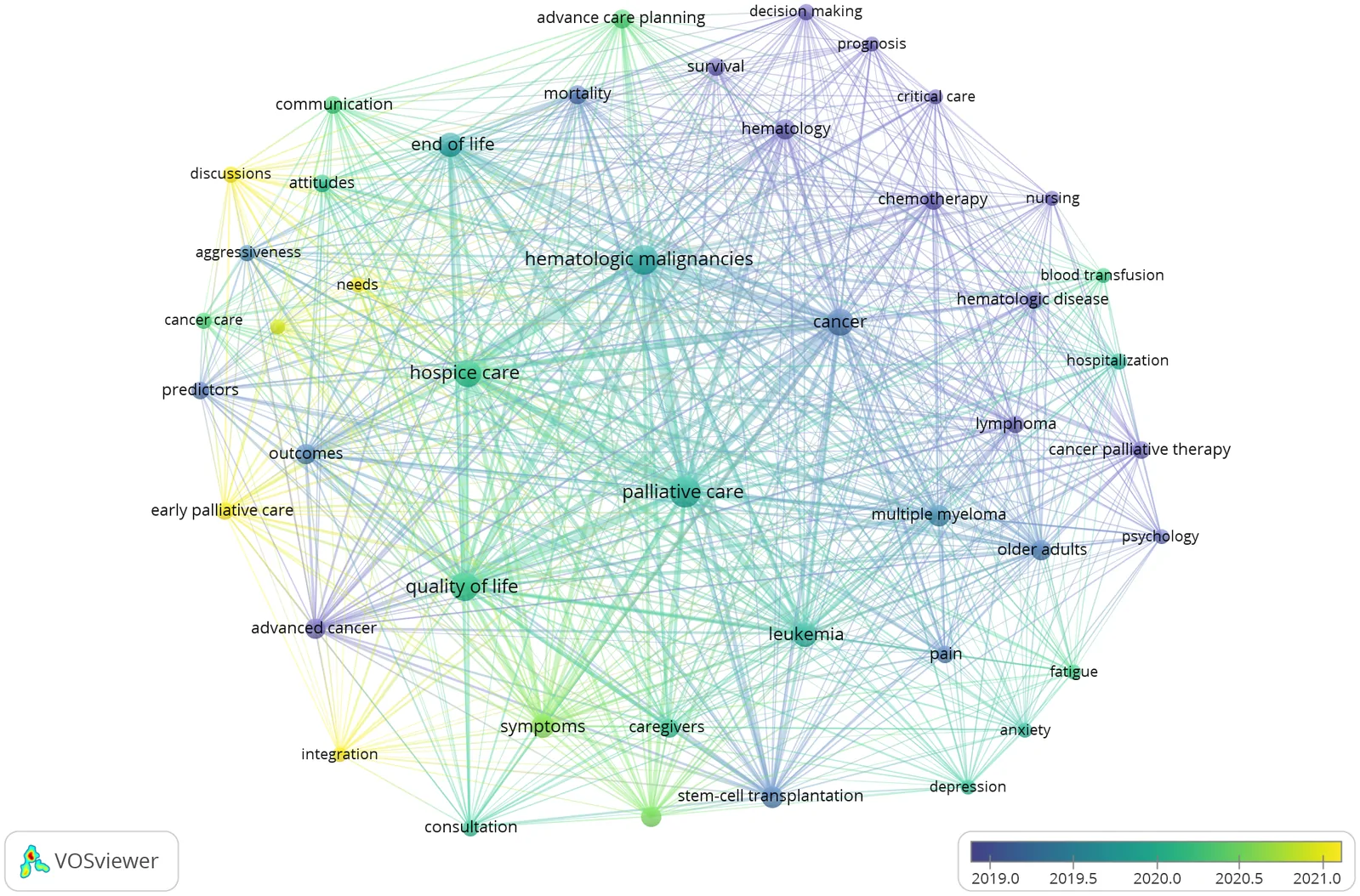
Keyword co-occurrence network (Overlay visualization). Node colors represent the average publication year of records containing each keyword, illustrating the temporal evolution of research themes.

Trend topic analysis traced the temporal evolution of research themes from 2002 to 2025 (**Figure 6**). The earliest topics (median years 2004-2010) centered on disease states (hematologic disease, chronic conditions, terminally ill, bone marrow failure, and hospital discharge), followed by symptom- and communication-oriented terms (end of life, pain management, communication-skills, psychological aspect) around 2011-2015. From 2016 onward, decision making, home, nursing, and stem-cell transplantation gained prominence. The most recent themes (median years 2021-2025) were quality of life, early palliative care, symptoms, discussions, and health care access, the last emerging only in 2024-2025.

**Figure 6:**
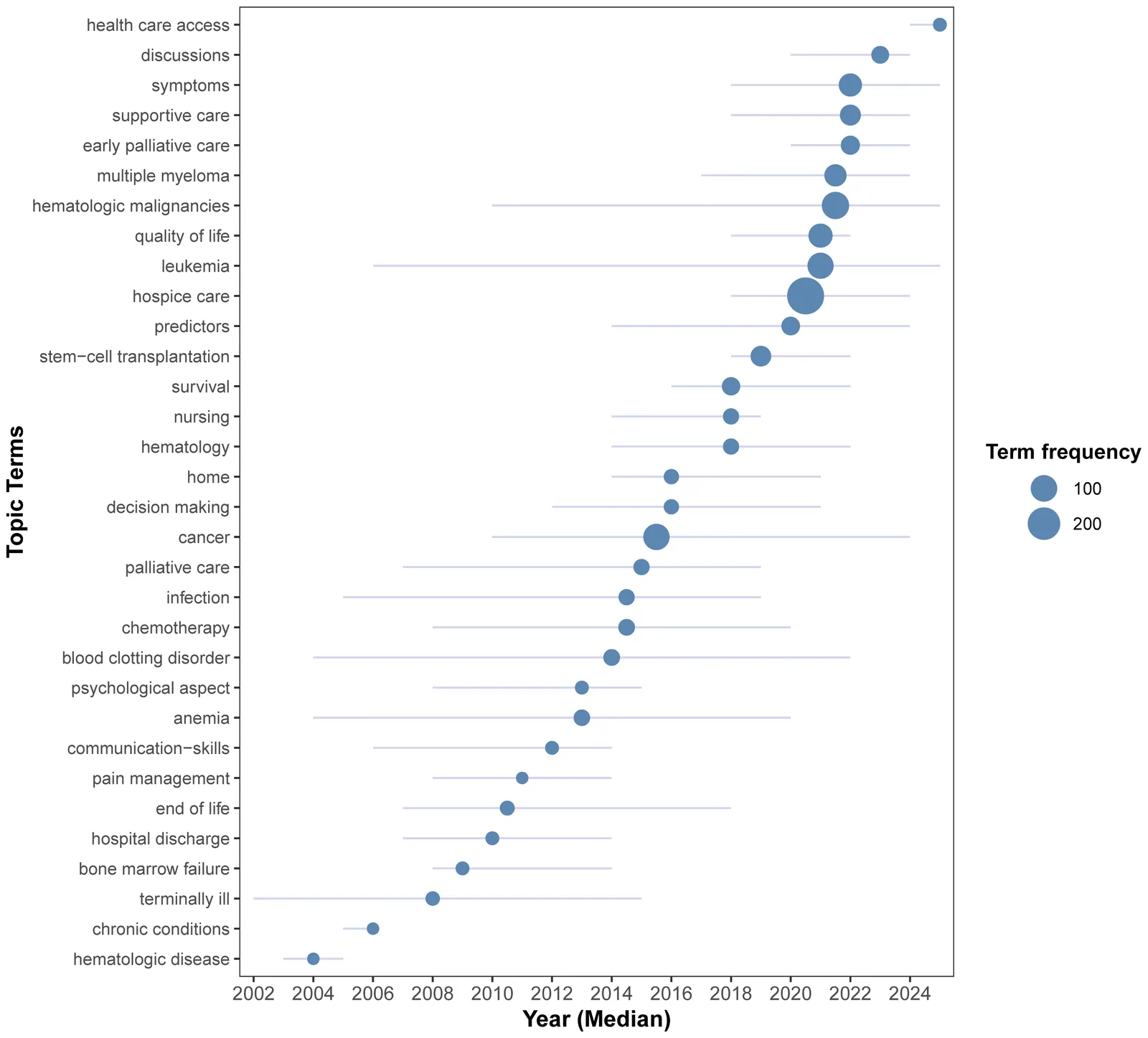
Publication-year distribution of trend topics. Topic terms occurring in at least two documents are shown. Points mark the document-level median publication year and point size represents document frequency.

The thematic evolution analysis identified seven communities in 2000-2013, four in 2014-2017, five in 2018-2021, and four in 2022-2025 (**Figure 7**; **Supplementary Table S4**). Early themes were fragmented across disease- and care-related topics, including quality of life/multiple myeloma/stem-cell transplantation, leukemia/older adults/mortality, chemotherapy/decision making/outcomes, and end of life/hospice care/home. These consolidated during 2014–2017 into four larger communities, notably hospice care/end of life/outcomes and quality of life/symptoms/supportive care. In 2018-2021, the largest communities centered on quality of life/hospice care/leukemia and end of life/hematology/attitudes. By 2022-2025, four communities remained: leukemia/end of life/multiple myeloma, hospice care/symptoms/supportive care, quality of life/stem-cell transplantation/outcomes, and older adults/chemotherapy/lymphoma.

**Figure 7:**
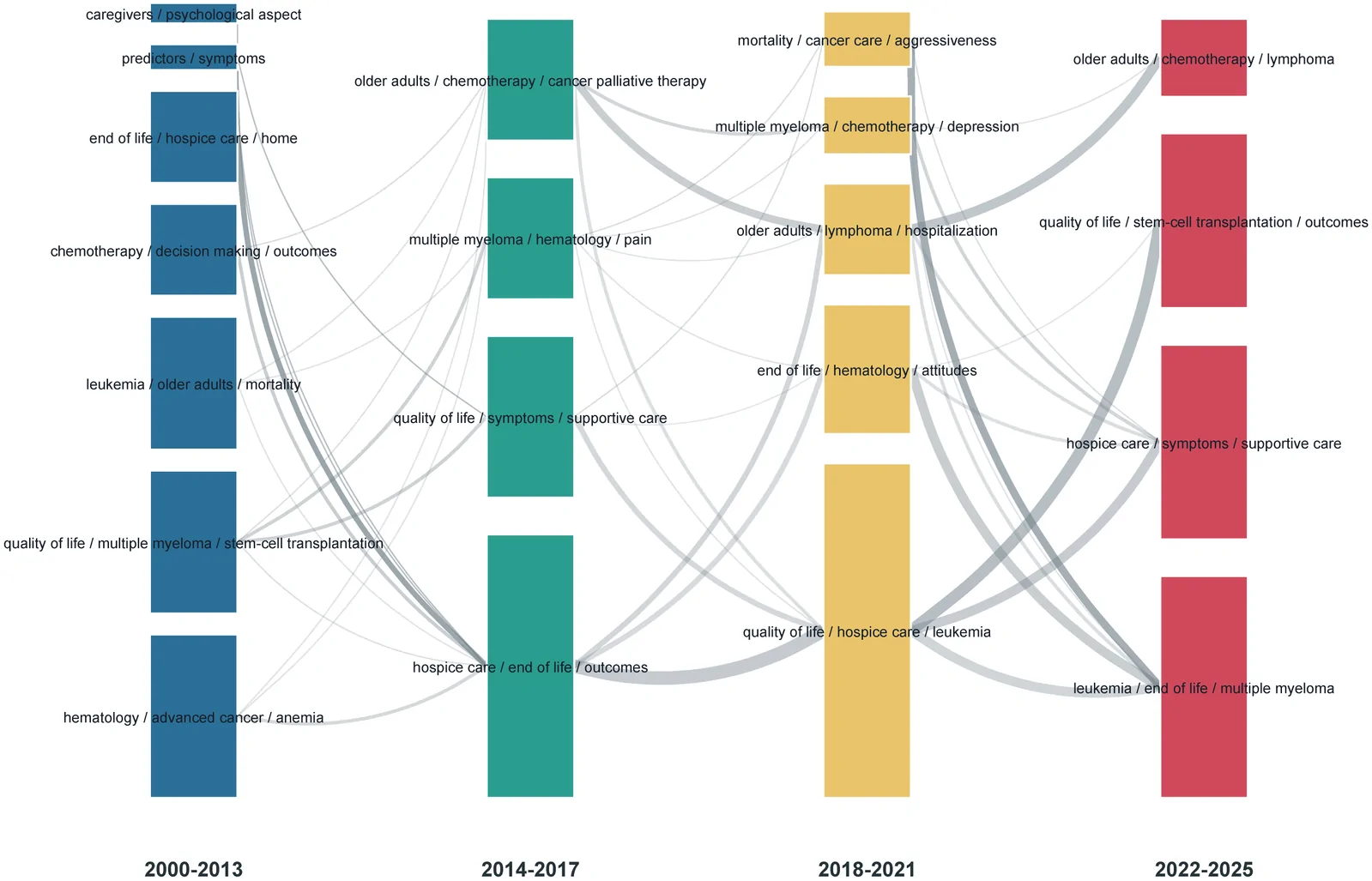
Thematic evolution of keywords across four publication periods. Binary document-level co-occurrence networks were constructed within 2000-2013, 2014-2017, 2018-2021, and 2022–2025 using up to 60 terms occurring in at least two documents. Rectangles represent Louvain communities, labels list leading constituent terms, and links connect adjacent-period communities that share terms; link width represents the extent of overlap.

Burst detection identified 30 keywords with significant bursts (**Figure 8**). The strongest bursts were observed for integration (strength 4.82, 2022-2024) and mortality (4.20, 2019-2021), followed by risk factor (2.63, 2020-2021) and end of life care (2.53, 2024). Temporally, three phases were evident. Early bursts (2017–2021) involved end-of-life terminology and antimyeloma treatment. Intermediate bursts (2020–2022) included risk factor, dignity therapy, and patient-reported outcome. The most recent bursts (2021 onward) centered on care delivery and patient experience—intensive chemotherapy, neurotoxicity, barriers, integration, symptoms, traumatic stress, advanced cancer, stem-cell transplantation, unmet needs—with end of life care, health care access, and equity and disparities still bursting through 2024-2025.

**Figure 8:**
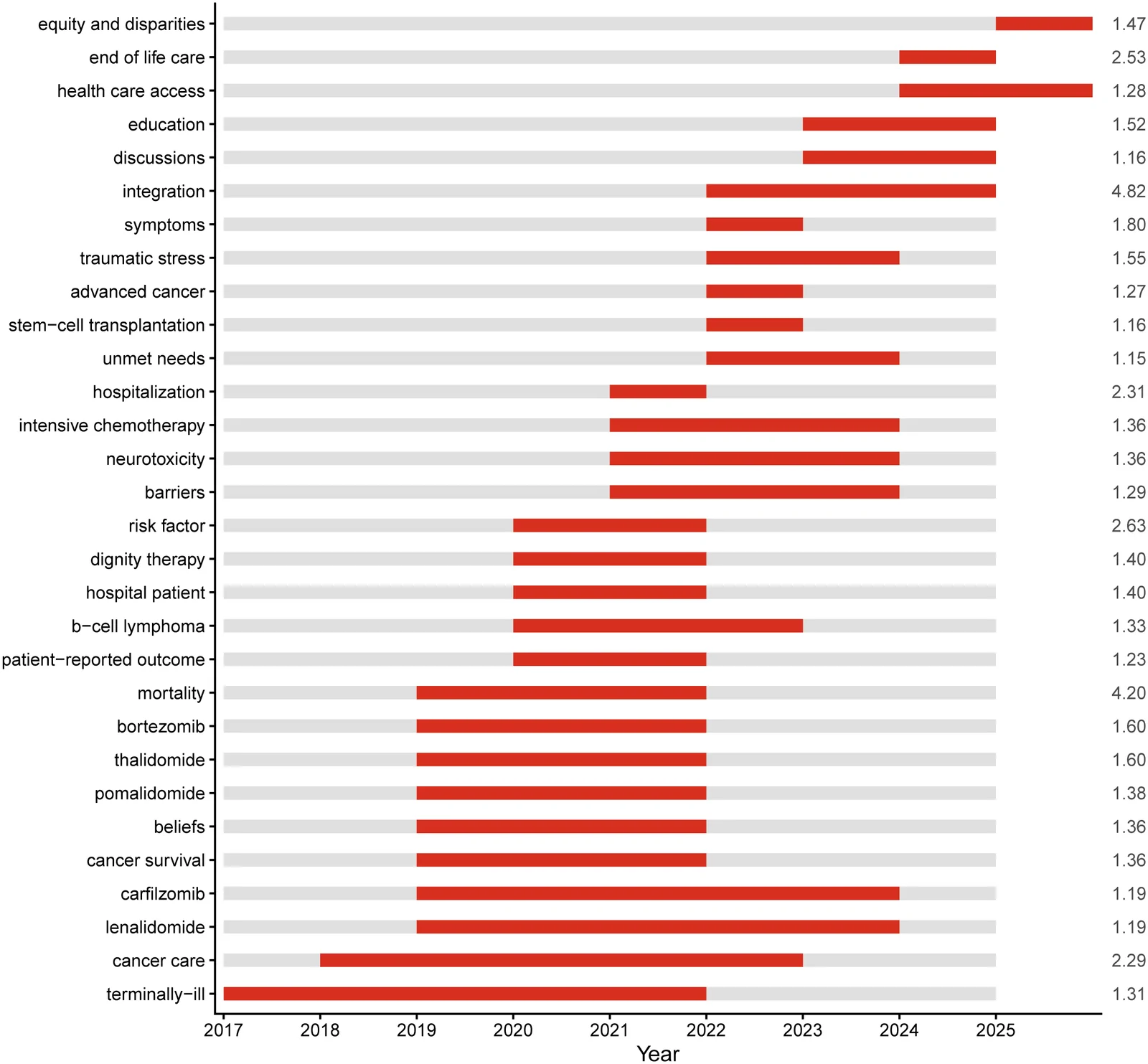
Thirty strongest algorithm-detected keyword bursts, 2017-2025. Burst detection used a Kleinberg-style finite-state model (s=2, γ=0.3) applied to yearly term counts normalized by annual publication output. Horizontal intervals show burst duration and numeric labels show burst strength.

### 3.4 Analysis of highly cited papers

The ten most locally cited publications received between 49 and 100 citations from other records in the corpus (**Table 5**). The highest-cited paper was Hui et al. (2014), which examined end-of-life care quality in patients with hematologic malignancies (31). The next most cited publication was the meta-analysis by Howell et al. (2011) on specialist palliative and hospice care referral (26). The focus-group study of hematologic oncologists’ end-of-life care decisions by Odejide et al. (2014) ranked third (11), followed by the HSCT inpatient palliative care trial by El-Jawahri et al. (2016) (4). The ten papers were published between 2010 and 2018, with three published in 2015. Journal of Pain and Symptom Management contributed three papers and Journal of Oncology Practice two. Their topics centered on three recurring concerns: end-of-life care quality and service use, symptom and referral needs, and integration of palliative care alongside active hematologic treatment. Several documents compared hematologic malignancies with solid tumors regarding service use, symptom needs, treatment near death, and referral practices (**Table 5**).

**Table 5:** Most locally cited papers under the topic of palliative care research in hematologic malignancies.

Paper	Journal	Year	Local citations	Key points
Quality of End-of-Life Care in Patients With Hematologic Malignancies [26]	Cancer	2014	100	This retrospective cohort study compared end-of-life care indicators between patients with hematologic malignancies and those with solid tumors. Patients with hematologic malignancies had more emergency visits, hospitalizations, intensive care unit admissions, cancer therapy near death, and lower palliative care unit use.
Haematological malignancy: are patients appropriately referred for specialist palliative and hospice care? A systematic review and meta-analysis of published data (26)	Palliative Medicine	2011	89	This review and meta-analysis examined referral to specialist palliative or hospice care among patients with hematological malignancies. It reported lower use of these services compared with other cancers and identified transitions to palliative care and referral barriers as research needs.
End-of-life care for blood cancers: A series of focus groups with hematologic oncologists [11]	Journal of Oncology Practice	2014	73	This qualitative study used four focus groups with 20 hematologic oncologists to explore end-of-life care decisions. Participants described difficulty recognizing the end-of-life phase, persistent curative expectations, barriers to end-of-life discussions, and limitations of home-based services. The findings highlighted the need for clearer clinical triggers and quality measures tailored to hematologic malignancies, including consideration of palliative transfusions.
Effect of Inpatient Palliative Care on Quality of Life 2 Weeks After Hematopoietic Stem Cell Transplantation A Randomized Clinical Trial (4)	JAMA	2016	72	This randomized clinical trial evaluated inpatient palliative care during hematopoietic stem cell transplantation. The intervention was associated with a smaller decline in quality of life at 2 weeks and less worsening of depression and symptom burden.
Perceptions of Palliative Care Among Hematologic Malignancy Specialists: A Mixed-Methods Study (9)	Journal of Oncology Practice	2015	62	This mixed-methods study compared perceptions of palliative care among hematologic and solid tumor oncologists. Hematologic oncologists more often viewed palliative care as end-of-life care and described specialty-specific barriers to referral.
Differences in attitudes and beliefs toward end-of-life care between hematologic and solid tumor oncology specialists (27)	Annals of Oncology	2015	53	This survey compared treatment recommendations and end-of-life care attitudes between hematologic and solid tumor oncology specialists. Hematologic specialists were more likely to favor systemic therapy near the end of life and reported lower comfort with death, dying, and hospice discussions.
Destined to die in hospital? Systematic review and meta-analysis of place of death in haematological malignancy (28)	BMC Palliative Care	2010	52	This systematic review identified 21 studies of place of death in adults with hematologic malignancies and included 17 in a meta-analysis. Compared with patients with other cancers, those with hematologic malignancies were more likely to die in hospital (odds ratio 2.25; 95% CI, 2.07-2.44). The study highlighted the need to understand patients' preferred place of care and address avoidable hospital deaths.
The Symptom Burden of Patients with Hematological Malignancy: A Cross-Sectional Observational Study (2)	Journal of Pain and Symptom Management	2011	50	This cross-sectional observational study assessed symptom burden in patients with hematological malignancy. Patients reported substantial physical and psychological symptoms, with higher burden among those on treatment, with poorer performance status, inpatients, and those with advanced disease.
What Is Different About Patients With Hematologic Malignancies? A Retrospective Cohort Study of Cancer Patients Referred to a Hospice Research Network (29)	Journal of Pain and Symptom Management	2015	50	This hospice-network cohort study compared patients with hematologic malignancies and solid tumors. Hematologic malignancy patients had poorer functional status, shorter hospice stays, and greater likelihood of death soon after hospice enrollment.
Comparing the Palliative Care Needs of Patients With Hematologic and Solid Malignancies (30)	Journal of Pain and Symptom Management	2018	49	This cross-sectional registry study compared unmet symptom needs at initial palliative care encounters. Patients with hematologic malignancies had symptom burdens comparable to those with solid tumors and higher odds of clinically significant tiredness and drowsiness.

### 3.5 Co-citation analysis

The reference co-citation network comprised 39 cited-reference nodes in three clusters (**Supplementary Figure S2**; **Supplementary Table S5**). Cluster 1 (red) contained 12 references and was organized around Hui et al. (2014) (31), Odejide et al. (2014) (11), and Hui et al. (2015) (27). Its references primarily addressed end-of-life care quality, treatment intensity, clinicians’ attitudes, and hospice-related barriers. Cluster 2 (blue) comprised 14 references and was centered on Temel et al. (2010) (8), Howell et al. (2011) (26), Manitta et al. (2011) (2), and Howell et al. (2010) (28). This cluster linked referral, symptom burden, and service needs with the broader oncology literature on early palliative care. Cluster 3 (green) contained 13 references, including El-Jawahri et al. (2016) (4), LeBlanc et al. (2015) (9), and Hochman et al. (2018) (30). Its references emphasized concurrent palliative care, psychological and quality-of-life outcomes, and communication.

Hui et al. (2014) had the highest PageRank (0.045) and betweenness centrality (30.254), followed in prominence by references including Howell et al. (2011), Temel et al. (2010), El-Jawahri et al. (2016), and LeBlanc et al. (2015) (**Supplementary Table S5**). Co-cited references were not restricted to documents included in the corpus, which explains the presence of general oncology studies such as Temel et al. (2010) (8).

### 3.6 Thematic strands in the recent substantive literature

The full-text synthesis identified five thematic strands in the recent substantive literature: transfusion access and hospice eligibility; advanced therapies and prognostic uncertainty; communication and primary palliative care capacity; equity and access; and caregiver burden (**Table 6**). These strands correspond to the most recent quantitative signals, including the 2024–2025 bursts for barriers, integration, health care access, and equity and disparities (**Figure 8**).

**Table 6:** Recent substantive literature grouped by thematic strand.

Thematic strand	Publication (ref. no.)	Journal, year	Design	Key contribution to the interpretation
Transfusion access and hospice eligibility	Odejide and Steensma (12)	Lancet Haematol, 2020	Review	Argued that transfusion-dependent patients should not be forced to choose between transfusion support and hospice care.
	LeBlanc et al. (13)	Blood, 2018	Retrospective cohort study	Transfusion dependence was associated with shorter hospice stays in leukemia, even though hospice enrollment was associated with less aggressive end-of-life care.
	Raman et al. (32)	JAMA Netw Open, 2025	Cross-sectional survey	Patients with blood cancers rated transfusion access as more important than routine hospice services.
	Potenza et al. (33)	J Pain Symptom Manage, 2025	Observational study	Early palliative care with goals-of-care conversations was associated with low-intensity end-of-life care in acute leukemia and high-risk MDS within a European outpatient model.
Advanced therapies and prognostic uncertainty	El-Jawahri et al. (34)	J Clin Oncol, 2025	Multisite randomized trial	Confirmed that inpatient palliative care during hematopoietic stem-cell transplantation improves quality of life and psychological outcomes.
	Connor Johnson et al. (35)	JNCCN, 2021	Retrospective cohort study	Documented frequent readmission, intensive care use, and intensive end-of-life care among CAR-T recipients.
	Stenson et al. (36)	Palliat Med, 2023	Qualitative service evaluation	Described persistent supportive care needs, prognostic uncertainty, and psychological distress among CAR-T recipients and caregivers.
	Tipian and Pandey (37)	Clin J Oncol Nurs, 2025	Practice report	Reported nursing-led early palliative care referral for patients receiving CAR-T therapy.
Communication and primary palliative care capacity	Hochrath et al. (3)	J Pain Symptom Manage, 2025	Systematic review	Found inadequate integration of primary palliative care in hematology.
	Tate et al. (38)	Palliat Support Care, 2025	Qualitative study	Identified facilitators and barriers to implementing advance care planning in hematologic malignancies.
	Potenza et al. (39)	Ann Hematol, 2025	National survey	Documented clinician-reported challenges and barriers to quality end-of-life care in hematologic malignancies.
Equity and access	Hsieh et al. (40)	Am J Hosp Palliat Care, 2025	Retrospective study	Reported lower palliative care use among Hispanic and African American patients, Medicaid-insured patients, and those admitted to small or rural hospitals.
	Ansah et al. (41)	Palliat Support Care, 2025	Scoping review	Mapped the limited evidence on palliative care provision for Black patients with hematologic cancers.
Caregiver burden	Alnaeem et al. (42)	Psychol Health Med, 2025	Cross-sectional survey	Documented substantial unmet needs, high burden, and poor quality of life among family caregivers of patients with advanced hematologic malignancy.

MDS, myelodysplastic syndromes; CAR-T, chimeric antigen receptor T-cell.

## 4 Discussion

### 4.1 Principal findings and conceptual contribution

This bibliometric review maps 307 publications on palliative care in hematologic malignancies indexed in the WoSCC and Scopus (2000–2025). Output grew at a descriptive annual rate of 17.6% and accelerated after 2017, yet knowledge production remained concentrated in a small number of North American and Western European academic groups, with only 14.98% international co-authorship. Dissemination was concentrated in palliative care venues: seven palliative, supportive care, or hospice journals carried 38.8% of the corpus, whereas the leading hematology journals carried only 19 publications. Four convergent temporal analyses (the keyword overlay, trend topics, thematic evolution, and burst detection) traced a conceptual progression from disease-directed palliation and end-of-life care toward early, concurrent palliative care integrated with intensive treatment. Integration, health care access, and equity and disparities formed the most recent fronts. Whereas narrative and systematic reviews describe intervention components and clinical effectiveness (1, 3), this analysis delineates the field’s temporal order, citation architecture, and thematic coupling.

### 4.2 From palliative treatment to a field of concurrent care

Early publications were few and fragmented. Within the retained corpus, the earliest influential work addressed symptom burden (2), place of death (28), delayed referral (43), and a “technological imperative” in which dying, prognostic understanding, and the desire for home death received little attention (44). From 2014, output expanded around end-of-life quality indicators (31), hospice utilization (45), hematologists’ perspectives (11), and advance care planning among transplant recipients and bereaved caregivers (46); after 2017, randomized studies of integrated palliative care in HSCT (47) and AML (48) reshaped the field. The current phase is implementation-oriented (the strongest keyword burst was integration, 2022-2024) and encompasses community-based pathways (49), goals-of-care communication (33, 50), palliative transfusion (32), caregiver needs (42), equitable access (40, 41), hospice prognostication (51), and palliative care coordination for chimeric antigen receptor T-cell (CAR-T) therapy recipients (37).

### 4.3 Two knowledge systems in collision

The keyword map separates a disease- and treatment-oriented vocabulary (multiple myeloma, lymphoma, chemotherapy, survival, mortality) from a palliative care vocabulary (quality of life, communication, advance care planning, hospice care). Hematology has traditionally organized attention around disease control and treatment intensity; palliative care organizes it around illness experience, prognostic uncertainty, and the meaning of treatment choices. The overlay visualization shows disease- and prognosis-related terms entering first, communication and advance care planning later, and early palliative care, integration, and needs most recently. The tightening links among HSCT, AML, quality of life, goals of care, and early palliative care mark a convergence of the two vocabularies. This convergence indicates that palliative care is increasingly studied while cure or remission remains possible.

The field’s knowledge base can be read at two levels. The most locally cited publications in the corpus converge on three concerns: the quality and intensity of end-of-life care (31), symptom burden and unmet referral needs (2, 26), and the demonstration that palliative care can be integrated alongside active hematologic treatment (4). Nearly all were published between 2010 and 2018, underscoring how recently this agenda crystallized. The co-citation network (**Supplementary Figure S2**) reaches deeper, exposing the foundations on which these works rest. Hui et al. (2014) occupies the most prominent bridging position in the network, linking the end-of-life quality literature to the wider field. The second cluster ties referral, symptom burden, and service needs (2, 26, 28) to the landmark solid-tumor early-palliative-care trials of Temel, Bakitas, and Zimmermann—indicating that the reorientation toward early integration was imported in part from general oncology. The third cluster, anchored by the HSCT trial of El-Jawahri et al. (4), consolidates concurrent palliative care as a distinct research program. Complementary work by Epstein, LeBlanc, Hui, and Odejide supplies the attitudinal evidence connecting these strands, showing how hematologists’ misconceptions and concerns about hope shape referral behavior (9, 10, 27, 52).

### 4.4 The gap between research enthusiasm and clinical uptake

A central tension in this map is the gap between academic enthusiasm for early integration and persistent clinical underuse. This characterization comes from the substantive literature: the ten most locally cited publications report late referral (26), short hospice stays (45), high hospital utilization and intensive treatment near death (31), and discomfort with goals-of-care discussions (11). Because citation accumulation favors older work, these studies necessarily describe an earlier state of care, and bibliometric data cannot establish whether the gap has since narrowed. The network does show that the field has begun to respond: barriers, integration, health care access, and equity and disparities were still bursting into 2024-2025.

Palliative transfusion is the clearest case. Many patients with advanced leukemia or marrow failure depend on blood transfusions for relief of fatigue, dyspnea, or bleeding risk, yet transfusion is difficult to access in many hospice systems and rarely available in home hospice. The consequences are measurable: transfusion-dependent patients with leukemia have markedly shorter hospice stays even though enrollment is associated with less aggressive end-of-life care and lower spending (13), and patients themselves rate transfusion access as more important than routine hospice services (32). This barrier operates at two levels. At the system level, it is most acute where hospice benefit structures effectively require forgoing transfusion support, as in the United States; in parts of Europe, home-based palliative care models partially decouple symptom support from hospice enrollment (33), underscoring the need for cross-system comparative research. At the conceptual level, transfusion is often construed as life-prolonging rather than symptom-relieving and therefore as misaligned with the philosophy of hospice care (12, 32). Durable solutions must address both levels. They should include payment, logistical, and home- or clinic-based models that permit palliative transfusion alongside hospice-level care, as well as closer hematology-hospice collaboration to align eligibility policies with the symptom-relieving intent of transfusion (12).

### 4.5 Advanced therapies and the need to embrace prognostic uncertainty

Trials in HSCT provided the disease-specific evidence that palliative care is compatible with intensive, potentially curative treatment: inpatient palliative care during transplantation attenuated declines in quality of life and lessened depression, anxiety, and post-traumatic stress symptoms (4, 47), findings confirmed in a 2025 multisite randomized trial (34). This evidence exemplifies, within hematology, the World Health Organization’s 2002 redefinition of palliative care as applicable early in the illness course and alongside life-prolonging therapy (53), and it aligns with the 2024 ASCO guideline recommending early, concurrent specialist palliative care (5). Uptake remains difficult, however, because treatment may retain curative potential late in the trajectory and because palliative care is still misconstrued as synonymous with end-of-life or hospice care. This misconception is held by hematologists (9–11) and by patients and family caregivers, for whom the term can evoke death, hopelessness, and abandonment of treatment (54).

CAR-T therapy intensifies this dilemma, and the recent bursts for intensive chemotherapy, neurotoxicity, and traumatic stress indicate that the research frontier is moving in the same direction. Recipients may hold realistic hope of a durable response while facing life-threatening, rapidly evolving complications (55). They experience substantial readmission, intensive care use, and intensive end-of-life care (35), and they report persistent supportive care needs, prognostic uncertainty, and psychological distress (36). Early values discussions and palliative care coordination appear feasible in CAR-T clinics, although patient-level outcome evidence remains limited (37). The clinical task is the timely introduction of palliative care that preserves legitimate hope, prepares families for rapid deterioration, and responds to symptoms that can change within days.

### 4.6 Hidden gaps revealed by the bibliometric map

First, research on specialist-delivered palliative care has outpaced research on the primary palliative care capacity of hematology teams. Specialist-oriented keywords (early palliative care, consultation, integration) are prominent; no generalist palliative care term reached the mapping threshold, and the intervention literature is concentrated in a few expert academic groups (**Table 1**). Because demand will always exceed specialist supply, sustainable integration requires hematologists to deliver primary palliative care with specialist support for complex needs (56). A recent systematic review likewise found inadequate integration of primary palliative care in hematologic malignancies (3), indicating that this gap is clinical as well as bibliometric. Second, communication research has expanded (38, 39). Clinicians remain uncertain about the timing and language of future-care discussions, which must acknowledge prognostic uncertainty while preserving hope.

Third, equity has only recently become visible—through bursts for equity and disparities and health care access into 2024–2025 and 2025 studies of racial disparities in palliative care use (40, 41). Equity also has a geographic dimension: the corpus is dominated by urban academic centers in North America and Western Europe, with weak collaboration links to Asian research groups, while the wider cancer literature shows that rurality is associated with more acute care near death and lower hospice use (57). People in rural and remote regions are thus largely absent from the evidence base, and implementation models must be adapted to health systems with fewer palliative care clinicians and different cultural expectations. Fourth, caregiver research remains underdeveloped relative to the documented burden. Caregivers recur in the thematic structure from 2000–2013 onward, yet only 27 documents carried the keyword, and caregiver-focused interventions are far less visible than patient-focused trials. This gap persists despite substantial unmet needs, high burden, and poor quality of life among family caregivers (42), whose capacity underpins home care, hospice use, and recovery after advanced therapies.

### 4.7 Implications for research and practice

These findings support framing palliative care in hematologic malignancies as concurrent serious illness care throughout the disease trajectory, particularly during HSCT or CAR-T therapy and in relapsed or refractory disease. Palliative transfusion should be prioritized in policy and implementation research (feasibility, symptom effects, bleeding-risk management, utilization, and payment mechanisms across health systems), and outcome frameworks should extend beyond quality of life to goal-concordant care, caregiver outcomes, time at home, and transfusion access.

The dissemination pattern suggests a further lever: most of this literature appears in palliative care journals rather than the hematology journals read by the clinicians best positioned to initiate early integration (**Table 3**). Whether this venue gap reflects submission choices, journal scope, or editorial factors cannot be determined bibliometrically, but greater visibility in hematology venues and more joint hematology-palliative care authorship could help narrow the divide. Multicenter, international, and community-based studies are likewise needed to broaden an evidence base concentrated in a few countries and author groups. Publication counts and co-occurrence networks indicate where little has been published; the clinical priority assigned here to equity, caregiver support, and transfusion access derives from the substantive literature reviewed above.

### 4.8 Limitations

There were several limitations. First, citation-based indicators reflect database coverage, publication age, indexing practices, and author-network structure as much as scholarly influence, and they cannot measure clinical effectiveness or rigor. Second, publications from 2024 to 2025 have had little time to accumulate citations; we mitigated this limitation with burst detection and median-year trend analyses rather than age-normalized citation indicators. Third, the restriction to English-language articles and reviews in WoSCC and Scopus has substantive consequences. Nursing, qualitative, and psychosocial research indexed primarily in MEDLINE or CINAHL is likely underrepresented, which probably skews the thematic profile toward clinically oriented journals. Regional, non-English, and grey literature may also have been missed. Fourth, screening decisions of the two reviewers were not formally recorded at the time of the original screen, so a quantitative inter-rater agreement statistic could not be computed. Fifth, the most central keyword nodes reproduce the search terms themselves, so their prominence is partly circular. Co-occurrence, thematic evolution, and burst results also depend on threshold and harmonization choices, and the treatment-content screen involved judgments about treatment intent that other teams might apply differently. Finally, these analyses reveal patterns of terminology and association; they cannot establish causal relationships or clinical priorities. We therefore integrated the bibliometric findings with the substantive content of highly cited and recent studies, and the results should be read as a map of the field’s intellectual development rather than a substitute for formal evidence synthesis.

## 5 Conclusion

Across 307 publications indexed in the WoSCC and Scopus (2000–2025), research on palliative care in hematologic malignancies increased at a descriptive annual rate of 17.6%, with more publications appearing after 2017. Keyword co-occurrence and thematic mapping show that recent publications more frequently address concurrent palliative care alongside intensive treatments such as HSCT, as well as integration, health care access, equity, and disparities. The included literature also discusses late referral and intensive care near death, but the bibliometric data cannot establish the frequency or temporal trends of these practices. Publications and collaborations were concentrated in a limited number of North American and Western European institutions, and relatively few articles appeared in hematology journals. Further empirical research could examine underrepresented settings and topics, including rural and remote populations, caregiver support, primary palliative care within hematology, and barriers to services such as palliative transfusion. Because this analysis is based primarily on publication, citation, and keyword data, its findings describe the structure and topics of the literature. They do not address the effectiveness of clinical interventions or changes in clinical practice.

## Data Availability

The original contributions presented in the study are included in the article/**Supplementary Material**. Further inquiries can be directed to the corresponding author.
