# Supplementary material for: Palliative care in hematologic malignancies: a bibliometric review and visualization analysis from 2000 to 2025
Source: Front Oncol. 2026 Oct 16;16:1939505. doi: 10.3389/fonc.2026.1939505 (PMC13719424; doi:10.3389/fonc.2026.1939505)
Supplement: Supplementary file 1 [file SupplementaryFile1.docx]

Figure S1 Author collaboration network. The thresholded VOSviewer network illustrates collaboration and co-publication patterns among productive contributors. The analysis includes authors with a minimum of six publications. Node size reflects the number of publications, while link thickness represents the strength of co-authorship. Colors delineate distinct collaborative clusters.

Figure S2 Reference co-citation network. The analysis includes 39 highly co-cited references. Nodes represent individual cited references, and links indicate the frequency with which two references are cited together.

Table S1 Main bibliometric characteristics of the dataset.

| Indicator | Value |
| --- | --- |
| Publish year | 2001-2025 |
| Sources | 108 |
| Documents | 307 |
| Annual growth rate | 17.6% |
| Average citations per document | 26.74 |
| References | 11,798 |
| Authors | 1,615 |
| Co-authors per document | 6.85 |
| International co-authorship | 14.98% |
| Articles | 258 |
| Reviews | 49 |

Table S2 The top 10 most productive countries.

| Country | Publications | Citations | Citations/Publications |
| --- | --- | --- | --- |
| United States | 146 | 4,729 | 32.39 |
| United Kingdom | 28 | 1,208 | 43.14 |
| Canada | 21 | 521 | 24.81 |
| Australia | 20 | 722 | 36.10 |
| Italy | 20 | 253 | 12.65 |
| Germany | 19 | 297 | 15.63 |
| China | 14 | 289 | 20.64 |
| France | 14 | 182 | 13.00 |
| Spain | 11 | 174 | 15.82 |
| Japan | 9 | 78 | 8.67 |

Table S3 Thematic communities and constituent terms across four publication periods.

| Label | Terms | Display Order |
| --- | --- | --- |
| hematology / advanced cancer / anemia | advanced cancer; anemia; blood clotting disorder; blood transfusion; cancer palliative therapy; chronic conditions; hematology; infection; methotrexate | 1 |
| quality of life / multiple myeloma / stem-cell transplantation | bone marrow failure; lymphoma; multiple myeloma; pain; quality of life; stem-cell transplantation; supportive care | 2 |
| leukemia / older adults / mortality | communication-skills; hospitalization; leukemia; mortality; myelodysplastic syndrome; older adults; place; statistical significance; young adult | 3 |
| chemotherapy / decision making / outcomes | chemotherapy; decision making; hospital discharge; medical decision making; outcomes; stem-cell transplantation | 4 |
| end of life / hospice care / home | end of life; home; hospice care; palliative care services | 5 |
| predictors / symptoms | predictors; symptoms | 6 |
| caregivers / psychological aspect | caregivers; psychological aspect | 7 |
| hospice care / end of life / outcomes | advanced cancer; attitudes; blood transfusion; bone-marrow-transplantation; caregivers; clinical decision making; consultation; decision making; discussions; end of life; ethics; health services; home; hospice care; illness trajectories; leukemia; lung-cancer; lymphoma; outcomes; predictors | 1 |
| quality of life / symptoms / supportive care | access; aggressiveness; burden; cell lung-cancer; clinical oncology; communication; functional status; hospital anxiety; multicenter; myelodysplastic syndromes; quality of life; supportive care; symptoms | 2 |
| multiple myeloma / hematology / pain | advance care planning; cancer pain; dyspnea; fatigue; hematology; mortality; multiple myeloma; pain; survival | 3 |
| older adults / chemotherapy / cancer palliative therapy | adolescent; cancer palliative therapy; chemotherapy; critical care; depression; hodgkin disease; lymphoma, non-hodgkin; nonhodgkin lymphoma; nursing; older adults; procedures; psychology; stem-cell transplantation | 4 |
| quality of life / hospice care / leukemia | advance care planning; advanced cancer; consultation; critical care; cross-sectional study; discussions; distress; early palliative care; elderly-patients; hospice care; leukemia; lung; outcomes; perceptions; predictors; quality of life; stem-cell transplantation; supportive care; symptoms | 1 |
| end of life / hematology / attitudes | advance directive; associations; attitudes; beliefs; caregivers; communication; dignity therapy; end of life; experiences; family; hematology; home-care; needs; perspectives; preferences | 2 |
| older adults / lymphoma / hospitalization | barriers; blood transfusion; cancer palliative therapy; cancer survival; decision making; hospitalization; lymphoma; nursing; older adults; patient referral; psychology; survival | 3 |
| multiple myeloma / chemotherapy / depression | antineoplastic agents; anxiety; bortezomib; carfilzomib; chemotherapy; depression; multiple myeloma; thalidomide | 4 |
| mortality / cancer care / aggressiveness | aggressiveness; cancer care; impact; mortality; place; prognosis | 5 |
| leukemia / end of life / multiple myeloma | advance care planning; aggressiveness; associations; attitudes; barriers; cancer care; communication; decision making; diagnosis; discussions; dying; early palliative care; education; end of life; intensity; leukemia; mortality; multiple myeloma; perspectives; predictors; prognosis | 1 |
| hospice care / symptoms / supportive care | anxiety; blood transfusion; critical care; depression; equity and disparities; hematology; hospice care; impact; needs; pain; perceptions; supportive care; survival; symptoms | 2 |
| quality of life / stem-cell transplantation / outcomes | access; advanced cancer; caregivers; consultation; end of life care; integration; lung; outcomes; quality of life; stem-cell transplantation; traumatic stress; unmet needs | 3 |
| older adults / chemotherapy / lymphoma | blood clotting disorder; cancer palliative therapy; charlson comorbidity index; chemotherapy; cohort analysis; comparative study; cyclophosphamide; fatigue; follow up; hospitalization; lymphoma; older adults; patient referral | 4 |

Table S4 Reference co-citation network metrics.

| Rank | Cited Reference Node | Paper Title | Cluster | Betweenness | Closeness | PageRank |
| --- | --- | --- | --- | --- | --- | --- |
| 1 | Hui D 2014 | Quality of End-of-Life Care in Patients With Hematologic Malignancies: A Retrospective Cohort Study | 1 | 30.254 | 0.045 | 0.045 |
| 2 | Odejide Oo 2014 | End-of-Life Care for Blood Cancers: A Series of Focus Groups With Hematologic Oncologists | 1 | 15.282 | 0.045 | 0.037 |
| 3 | El-Jawahri Ar 2015 | Health Care Utilization and End-of-Life Care for Older Patients With Acute Myeloid Leukemia | 1 | 9.360 | 0.045 | 0.030 |
| 4 | Hui D 2015 | Differences in Attitudes and Beliefs Toward End-of-Life Care Between Hematologic and Solid Tumor Oncology Specialists | 1 | 11.717 | 0.045 | 0.029 |
| 5 | Odejide Oo 2016-1 | Barriers to Quality End-of-Life Care for Patients With Blood Cancers | 1 | 4.470 | 0.045 | 0.027 |
| 6 | Leblanc Tw 2018 | Transfusion Dependence, Use of Hospice Services, and Quality of End-of-Life Care in Leukemia | 1 | 5.267 | 0.044 | 0.026 |
| 7 | Odejide Oo 2017 | Why Are Patients With Blood Cancers More Likely to Die Without Hospice? | 1 | 5.524 | 0.045 | 0.024 |
| 8 | Wang R 2017 | Health Care Use by Older Adults With Acute Myeloid Leukemia at the End of Life | 1 | 1.690 | 0.044 | 0.021 |
| 9 | Wright Aa 2008 | Associations Between End-of-Life Discussions, Patient Mental Health, Medical Care Near Death, and Caregiver Bereavement Adjustment | 1 | 1.091 | 0.045 | 0.015 |
| 10 | Sexauer A 2014 | Patterns of Hospice Use in Patients Dying From Hematologic Malignancies | 1 | 1.949 | 0.045 | 0.017 |
| 11 | Odejide Oo 2016-2 | Timeliness of End-of-Life Discussions for Blood Cancers: A National Survey of Hematologic Oncologists | 1 | 1.589 | 0.045 | 0.017 |
| 12 | Earle Cc 2008 | Aggressiveness of Cancer Care Near the End of Life: Is It a Quality-of-Care Issue? | 1 | 1.681 | 0.044 | 0.016 |
| 13 | Howell Da 2011 | Haematological Malignancy: Are Patients Appropriately Referred for Specialist Palliative and Hospice Care? A Systematic Review and Meta-Analysis of Published Data | 2 | 17.108 | 0.043 | 0.043 |
| 14 | Temel Js 2010 | Early Palliative Care for Patients With Metastatic Non-Small-Cell Lung Cancer | 2 | 15.855 | 0.043 | 0.042 |
| 15 | Howell Debra 2010 | Destined to Die in Hospital? Systematic Review and Meta-Analysis of Place of Death in Haematological Malignancy | 2 | 6.089 | 0.043 | 0.029 |
| 16 | Leblanc Tw 2015-2 | What Is Different About Patients With Hematologic Malignancies? A Retrospective Cohort Study of Cancer Patients Referred to a Hospice Research Network | 2 | 7.757 | 0.043 | 0.029 |
| 17 | Manitta V 2011 | The Symptom Burden of Patients With Hematological Malignancy: A Cross-Sectional Observational Study | 2 | 6.309 | 0.043 | 0.029 |
| 18 | Fadul Na 2008 | Comparison of Symptom Burden Among Patients Referred to Palliative Care With Hematologic Malignancies Versus Those With Solid Tumors | 2 | 3.332 | 0.043 | 0.027 |
| 19 | Manitta Vj 2010 | Palliative Care and the Hemato-Oncological Patient: Can We Live Together? A Review of the Literature | 2 | 2.188 | 0.043 | 0.024 |
| 20 | Epstein As 2012 | Palliative Care and Hematologic Oncology: The Promise of Collaboration | 2 | 3.287 | 0.043 | 0.025 |
| 21 | Bakitas M 2009 | Effects of a Palliative Care Intervention on Clinical Outcomes in Patients With Advanced Cancer: The Project ENABLE II Randomized Controlled Trial | 2 | 4.329 | 0.043 | 0.028 |
| 22 | Bakitas Ma 2015 | Early Versus Delayed Initiation of Concurrent Palliative Oncology Care: Patient Outcomes in the ENABLE III Randomized Controlled Trial | 2 | 4.806 | 0.043 | 0.025 |
| 23 | Zimmermann C 2014 | Early Palliative Care for Patients With Advanced Cancer: A Cluster-Randomised Controlled Trial | 2 | 4.284 | 0.043 | 0.025 |
| 24 | Fadul N 2007 | Predictors of Access to Palliative Care Services Among Patients Who Died at a Comprehensive Cancer Center | 2 | 1.211 | 0.043 | 0.018 |
| 25 | Zimmermann C 2013 | Symptom Burden and Supportive Care in Patients With Acute Leukemia | 2 | 1.487 | 0.043 | 0.018 |
| 26 | Mcgrath P 2007 | Special Considerations for Haematology Patients in Relation to End-of-Life Care: Australian Findings | 2 | 0.483 | 0.042 | 0.013 |
| 27 | El-Jawahri A 2016 | Effect of Inpatient Palliative Care on Quality of Life 2 Weeks After Hematopoietic Stem Cell Transplantation: A Randomized Clinical Trial | 3 | 14.086 | 0.044 | 0.038 |
| 28 | Leblanc Tw 2015-1 | Perceptions of Palliative Care Among Hematologic Malignancy Specialists: A Mixed-Methods Study | 3 | 13.281 | 0.044 | 0.033 |
| 29 | Hochman Mj 2018 | Comparing the Palliative Care Needs of Patients With Hematologic and Solid Malignancies | 3 | 6.672 | 0.044 | 0.028 |
| 30 | Leblanc Tw 2015-3 | When and Why Should Patients With Hematologic Malignancies See a Palliative Care Specialist? | 3 | 8.069 | 0.044 | 0.028 |
| 31 | El-Jawahri A 2021 | Effectiveness of Integrated Palliative and Oncology Care for Patients With Acute Myeloid Leukemia: A Randomized Clinical Trial | 3 | 3.434 | 0.044 | 0.024 |
| 32 | El-Jawahri A 2020 | Palliative and End-of-Life Care for Patients With Hematologic Malignancies | 3 | 3.201 | 0.044 | 0.024 |
| 33 | El-Jawahri A 2017 | Effect of Inpatient Palliative Care During Hematopoietic Stem-Cell Transplant on Psychological Distress 6 Months After Transplant: Results of a Randomized Clinical Trial | 3 | 4.618 | 0.043 | 0.027 |
| 34 | Ferrell Br 2017 | Integration of Palliative Care Into Standard Oncology Care: American Society of Clinical Oncology Clinical Practice Guideline Update | 3 | 3.207 | 0.044 | 0.022 |
| 35 | Moreno-Alonso D 2018 | Palliative Care in Patients With Haematological Neoplasms: An Integrative Systematic Review | 3 | 2.195 | 0.044 | 0.018 |
| 36 | Leblanc Tw 2017 | Early Palliative Care for Patients With Hematologic Malignancies: Is It Really So Difficult to Achieve? | 3 | 2.914 | 0.044 | 0.020 |
| 37 | Beaussant Y 2018 | Hospital End-of-Life Care in Haematological Malignancies | 3 | 3.326 | 0.044 | 0.020 |
| 38 | Mccaughan D 2018 | Palliative Care Specialists' Perceptions Concerning Referral of Haematology Patients to Their Services: Findings From a Qualitative Study | 3 | 2.937 | 0.044 | 0.019 |
| 39 | Porta-Sales J 2017 | Is Early Palliative Care Feasible in Patients With Multiple Myeloma? | 3 | 1.660 | 0.044 | 0.018 |

Cluster indicates the community assignment in the reference co-citation network. Betweenness is a centrality measure reflecting the extent to which a cited reference lies on the shortest paths between other cited references; higher values indicate a stronger bridging position. Closeness measures how near a cited reference is to all other cited references in the network based on shortest path distance; higher values indicate greater overall proximity. PageRank is a link-based centrality measure reflecting the relative importance of a cited reference according to its connections and the importance of connected references; higher values indicate greater network prominence.
